# Supplementary material for: ROR2 Is Epigenetically Regulated in Endometrial Cancer
Source: Cancers (Basel). 2021 Jan 21;13(3):383. doi: 10.3390/cancers13030383 (PMC7864349; doi:10.3390/cancers13030383)
Supplement: Supplementary file 1 [file cancers-13-00383-s001.pdf]

cancers

Supplementary Materials: ROR2 is Epigenetically Regulated in Endometrial Cancer

Dongli Liu, Luis Enriquez and Caroline E. Ford

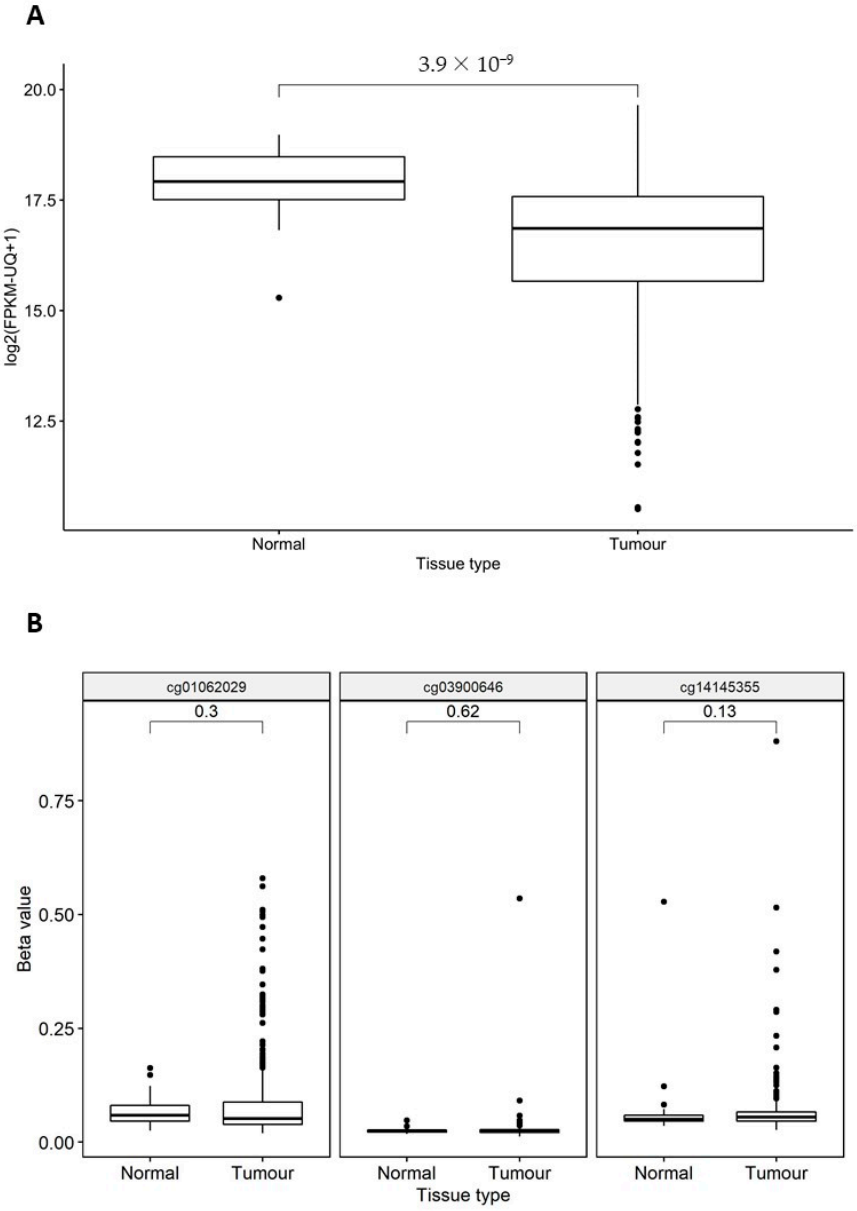

Figure S1. ROR2 was expressed significantly lower in tumour compared to normal tissue in the TCGA-UCEC cohort. (**A**). ROR2 expression level was significantly different between normal and tumour tissue of endometrial cancer. (**B**). No significant difference of methylation level in any of the three probes was observed between normal and tumour tissue.

Citation: Liu, D.; Enriquez, L.; Ford, C.E. ROR2 is Epigenetically Regulated in Endometrial Cancer. *Cancers* **2021**, *13*, 383. https://doi.org/10.3390/cancers13030383

Received: 9 December 2020
Accepted: 18 January 2021
Published: 21 January 2021

Publisher's Note: MDPI stays neutral with regard to jurisdictional claims in published maps and institutional affiliations.

Copyright: © 2021 by the authors. Licensee MDPI, Basel, Switzerland. This article is an open access article distributed under the terms and conditions of the Creative Commons Attribution (CC BY) license (http://creativecommons.org/licenses/by/4.0/).

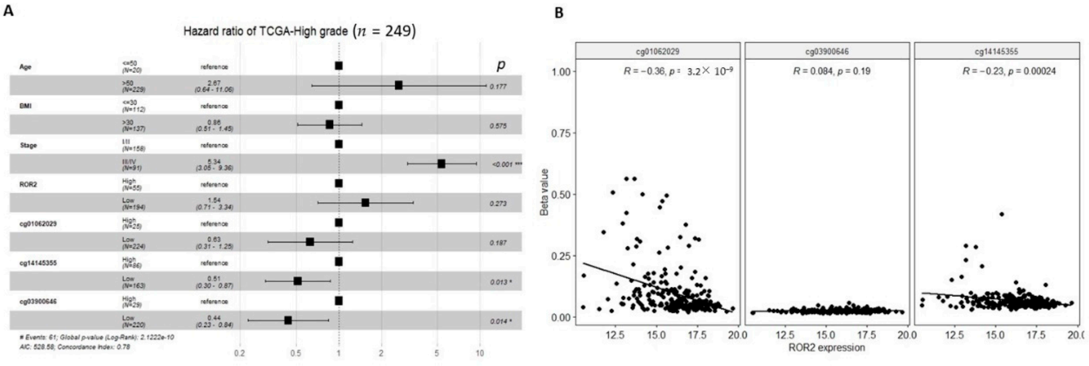

Figure S2. Multivariable overall survival and correlation analysis of ROR2 in high-grade subgroup of TCGA-UCEC cohort. (**A**). Methylation levels of cg14145355 and cg03900646 on ROR2 promoter as well as stage were significantly associated with overall survival in high grade patients of TCGA-UCEC cohort. (**B**). Methylation level of cg01062029 on ROR2 promoter as well as stage significantly associated with overall survival in serous patients of TCGA-UCEC cohort.

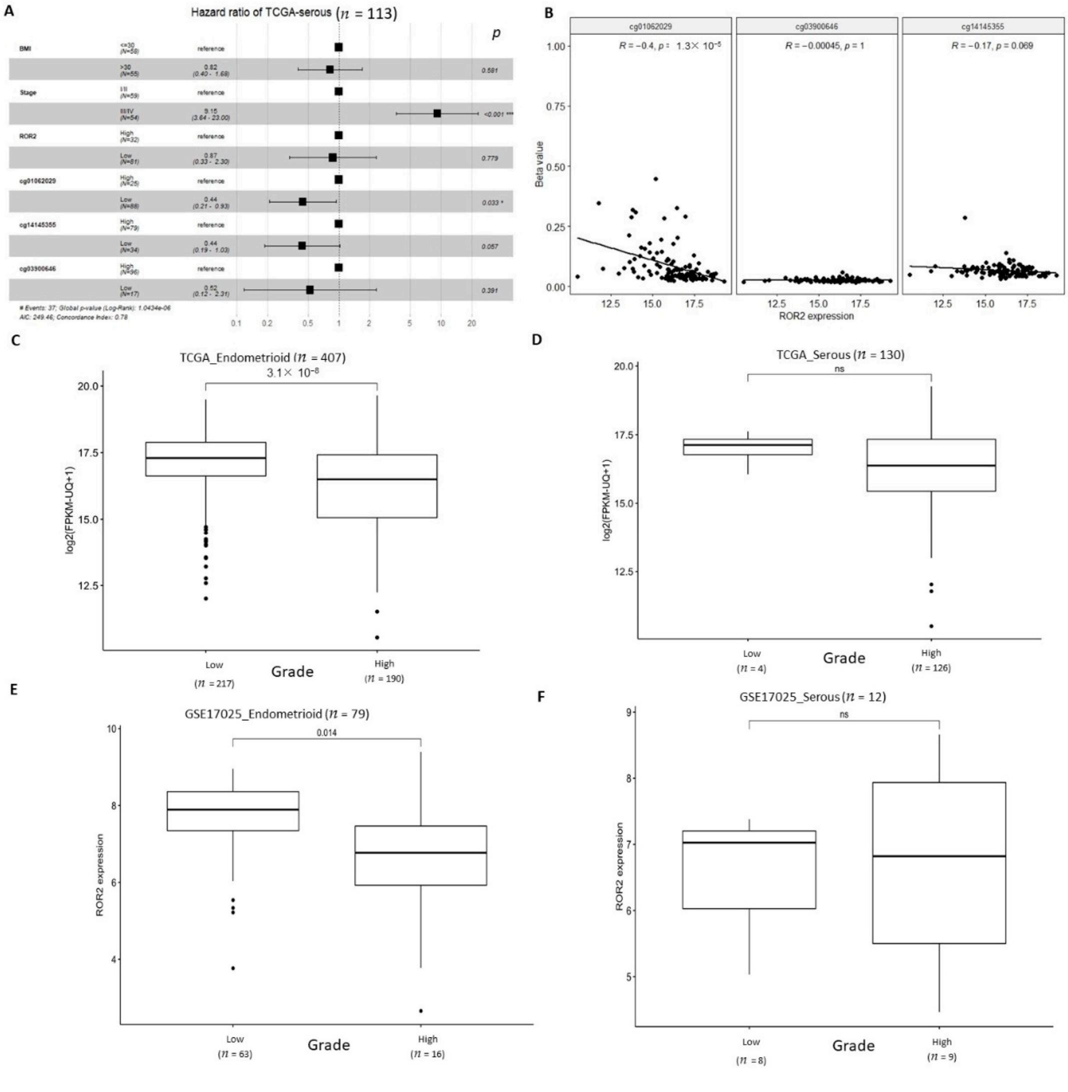

Figure S3. Multivariable overall survival and correlation analysis of ROR2 in subgroups stratified by histology subtypes of TCGA-UCEC and GSE17025 cohorts. (**A**). *ROR2* expression was negatively correlated with methylation level at cg01062029 and cg14145335 sites in high grade subgroup of TCGA-UCEC cohort (p < 0.001). (**B**). ROR2 expression was negatively correlated with methylation level at cg01062029 site in high grade subgroup of TCGA-UCEC cohort (p < 0.001).

(**C**). *ROR2* expression was significantly different between different grades in the subgroup of endometrioid endometrial cancer of TCGA-UCEC. (**D**). No significant difference was observed between different grades in serous endometrial cancer of TCGA-UCEC. (**E**). ROR2 expression was significantly different between different grades in the endometrioid endometrial cancer of GSE17025. (**F**). No significant difference was observed between different grades in serous endometrial cancer of GSE17025. ns. Not significant at $p < 0.05$ level.

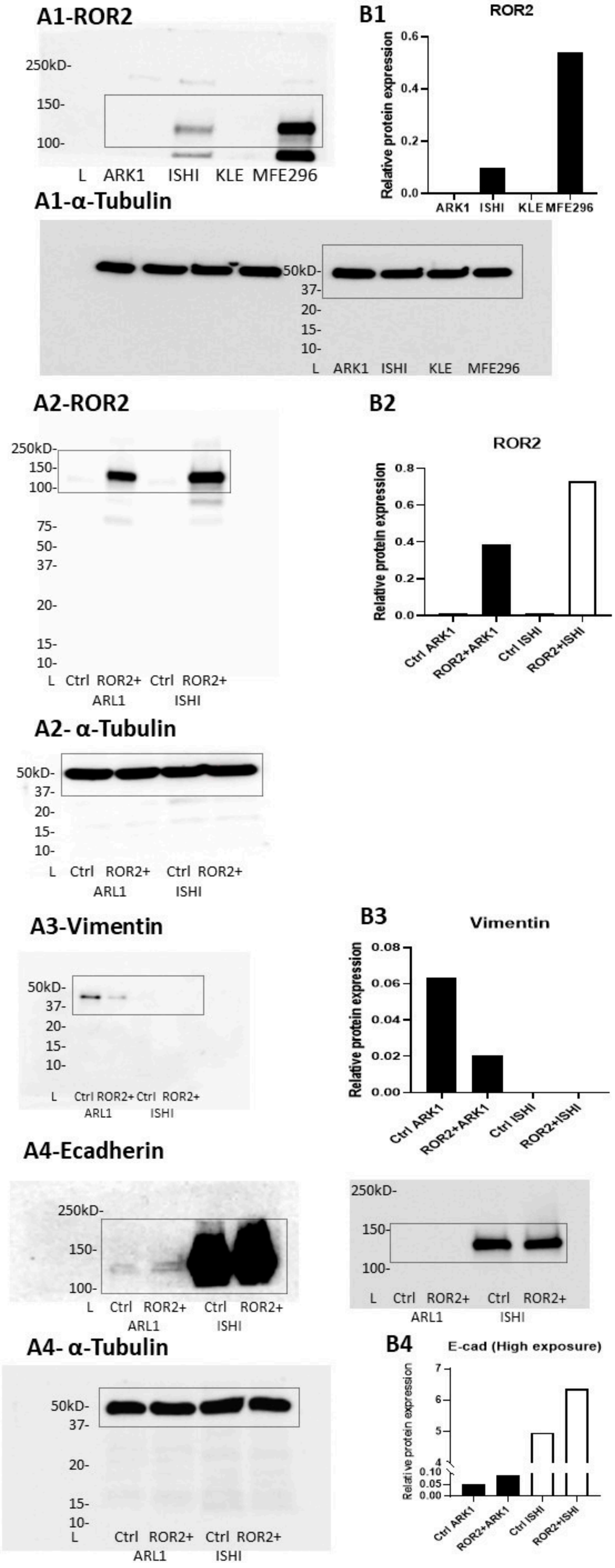

Figure S4. Relative protein expression level and uncropped pictures of western blotting membranes of the study. For each blot, chemiluminescence and epi-illuminated acquisitions were performed simultaneously using the ImageQuant LAS4000 system (GE Healthcare, USA). Blots

shown in Figure 4D were extracted from blots A1. Blots shown in Figure 6 were extracted from blots A2-A4. B: The intensity of the targets was quantified with the ImageQuantTL software and normalised against α-tubulin intensities. The relative expression level of the protein of interest was presented in the corresponding bar chart.

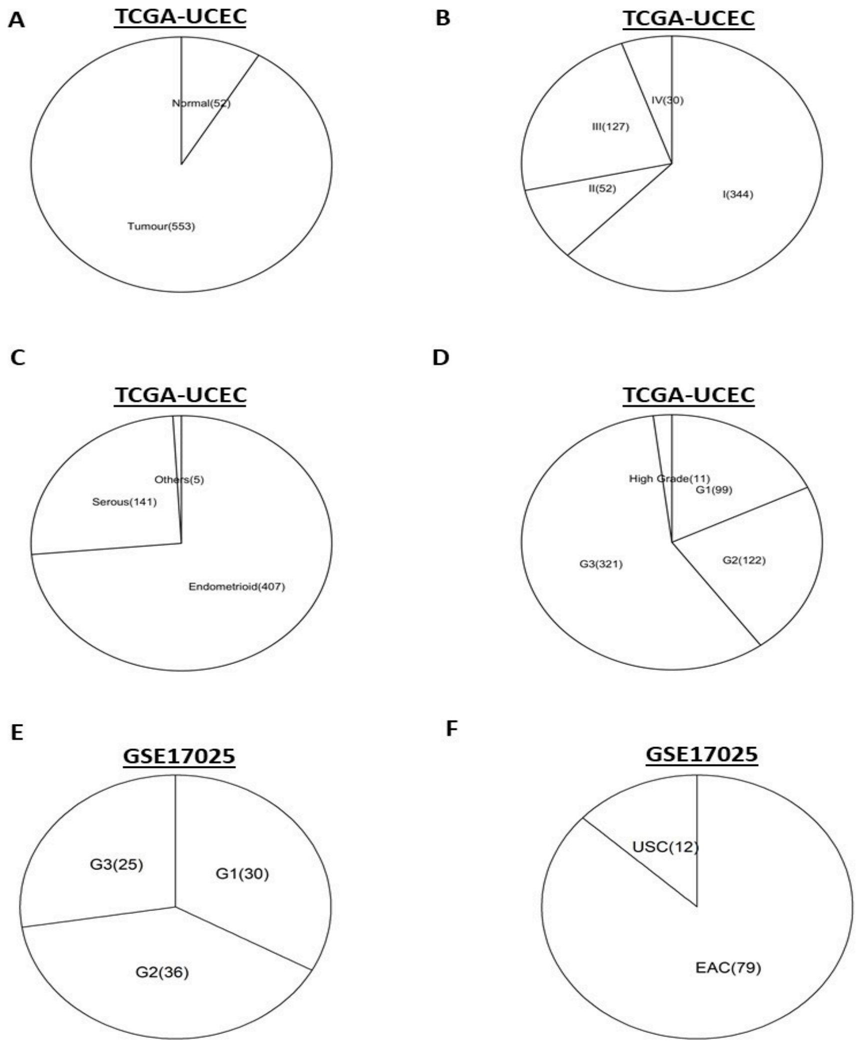

Figure S5. Distribution of the clinicopathological, gene expression and two methylation probes data from the TCGA-UCEC and GSE17025 datasets. (**A**). Frequency of normal tissue and tumour tissue included in the TCGA-UCEC cohort. (**B**–**D**). Distribution of stage, histological subtypes and grade from the tumour tissue of the TCGA-UCEC cohort. (**E**,**F**). Distribution of grade and subtypes of the GSE17025 cohort. EAC- Endometrioid adenocarcinoma of endometrium, USC- Uterine serous carcinoma.

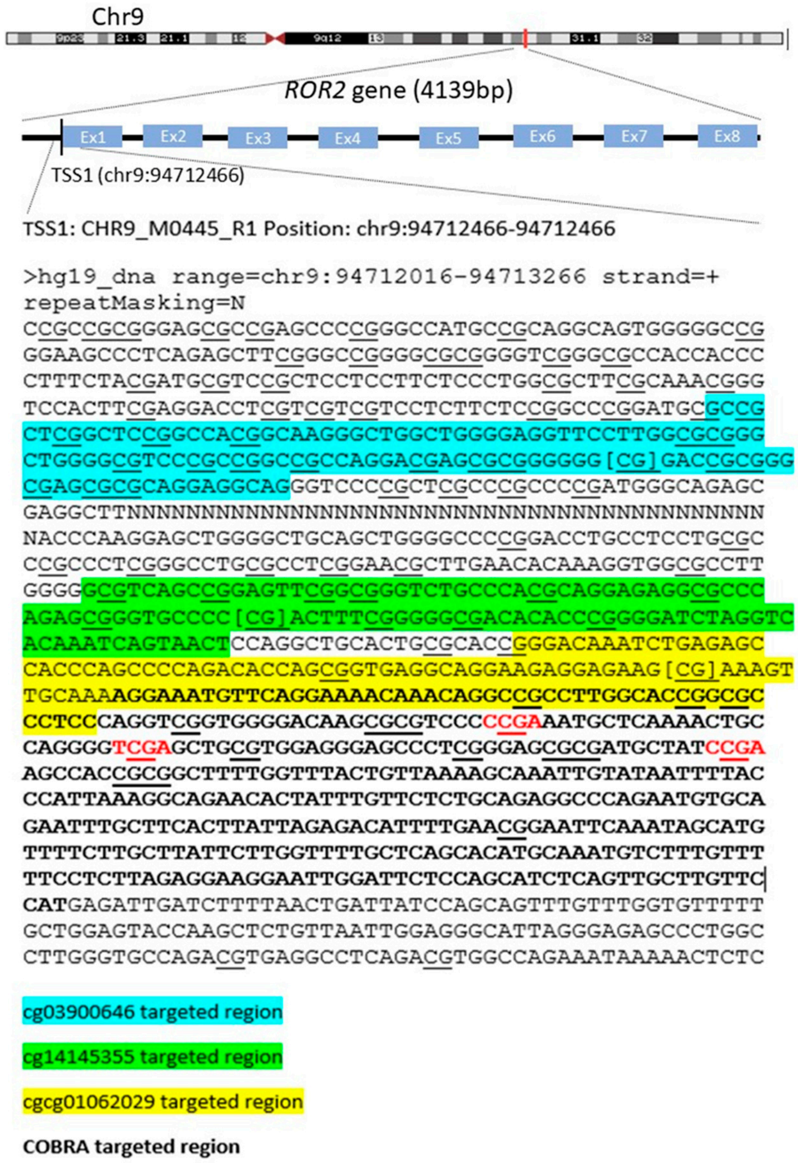

Figure S6. The schematic diagram of the targeted regions on ROR2 gene. TaqI restriction sites were highlighted with red.
